# Responses to Common Misconceptions Relating to COVID-19 Variant-Adapted mRNA Vaccines

**DOI:** 10.3390/vaccines12010057

**Published:** 2024-01-06

**Authors:** George Kassianos, Pauline MacDonald, Ivan Aloysius, Shanti Pather

**Affiliations:** 1 Royal College of General Practitioners, London NW1 2FB, UK; gckassianos@icloud.com; 2 British Global and Travel Health Association, London NW1 2FB, UK; 3 Infection Matters Ltd., London WC2H 9JQ, UK; macdonald@infectionmatters.com; 4 Ringmead Medical Group, Bracknell RG12 7WW, UK; i.aloysius@nhs.net; 5 BioNTech, An der Goldgrube 12, 55131 Mainz, Germany

**Keywords:** COVID-19, SARS-CoV-2, BNT162b2, mRNA vaccines, SARS-CoV-2 Omicron variant, vaccination hesitancy

## Abstract

The evolution of severe acute respiratory syndrome coronavirus 2 (SARS-CoV-2) and the waning of immunity over time has necessitated the use of booster doses of original coronavirus disease 2019 (COVID-19) vaccines. This has also led to the development and implementation of variant-adapted messenger RNA (mRNA) vaccines that include an Omicron sub-lineage component in addition to the antigen based on the wild-type virus spike protein. Subsequent emergence of the recombinant XBB sub-lineages triggered the development of monovalent XBB-based variant-adapted mRNA vaccines, which are available for vaccination campaigns in late 2023. Misconceptions about new variant-adapted vaccines may exacerbate vaccine fatigue and drive the lack of vaccine acceptance. This article aims to address common concerns about the development and use of COVID-19 variant-adapted mRNA vaccines that have emerged as SARS-CoV-2 has continued to evolve.

## 1. Introduction

Vaccination hesitancy, defined as ‘the reluctance or refusal to vaccinate despite the availability of vaccines’, was already a concern before the coronavirus disease 2019 (COVID-19) pandemic, being named as one of the top 10 threats to global health by the World Health Organization (WHO) in 2019 [1]. During the pandemic, the lack of vaccine acceptance has been a growing phenomenon, impeding the effectiveness of disease control and prevention efforts, plus potentially having a substantial effect on the trajectory of the pandemic and beyond [2,3]. The accelerated development and authorization of COVID-19 vaccines, the spread of misinformation (false information spread regardless of intent) and disinformation (false information spread intentionally to mislead) regarding COVID-19 vaccines, as well as concerns about very rare post-vaccination adverse events (AEs) such as myocarditis, have contributed to a greater degree of questioning and anxiety around vaccination among the general population [4,5].

**Figure 1:**
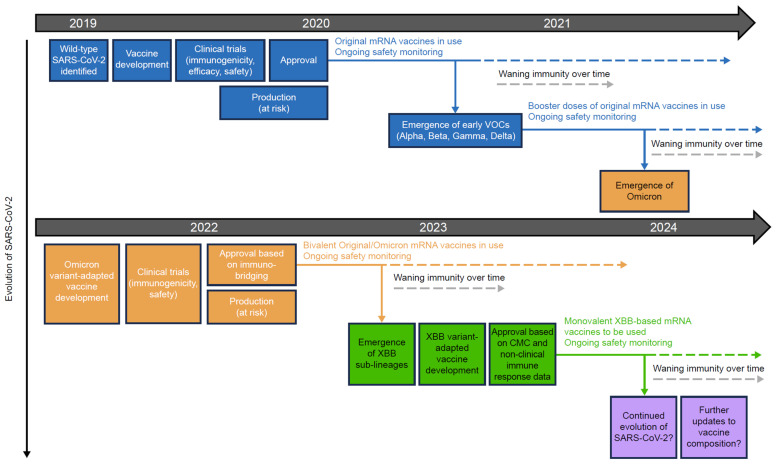
Development and approval process for original and variant-adapted COVID-19 mRNA vaccines. COVID-19, coronavirus disease 2019; CMC, chemistry, manufacturing, and control; mRNA, messenger RNA; SARS-CoV-2, severe acute respiratory syndrome coronavirus 2; VOC, variant of concern.

The evolution of severe acute respiratory syndrome coronavirus 2 (SARS-CoV-2) and waning of immunity has necessitated booster doses of COVID-19 vaccines (Figure 1). The emergence of the highly transmissible Omicron BA.1 variant of SARS-CoV-2 in November 2021 [6], and subsequent sub-lineages such as BA.4 and BA.5, all of which displayed pathological, genetic, and antigenic features that clearly distinguished them from prior SARS-CoV-2 variants of concern (VOCs) [7,8], led public health and regulatory authorities to advise vaccine manufacturers to develop bivalent COVID-19 vaccines that included Omicron sub-lineage spike protein components as well as the original wild-type virus spike protein (Figure 1) [9]. The continued evolution of SARS-CoV-2 subsequently resulted in the emergence of the XBB variant, a recombinant lineage derived from two BA.2 sub-lineages [10]. XBB descendent sub-lineages became dominant globally [11] and exhibited enhanced transmissibility and immuno-evasive properties compared with earlier variants [12]. This led to the development of monovalent XBB-containing vaccines for late-2023 vaccination campaigns (Figure 1) [13,14]. SARS-CoV-2 continues to evolve, with the emergence of EG.5, BA.2.86, and FL.1.5.1 [15,16]. Repeated vaccination campaigns with variant-adapted messenger RNA (mRNA) vaccines may contribute to further vaccine fatigue, as recipients may not understand the rationale for additional doses or appreciate their need.

Healthcare provider recommendations, particularly from primary healthcare professionals, strongly influence vaccine decision-making [1] and play a crucial part in combating vaccine anxiety and fatigue by effectively identifying and dispelling common vaccination myths using robust scientific evidence. This article aims to equip primary healthcare providers, and other relevant healthcare professionals, including pediatricians, with scientific evidence that can be used to address specific concerns regarding COVID-19 mRNA variant-adapted vaccines during communications with potential vaccine recipients. The article focuses on variant-adapted mRNA vaccines because original COVID-19 vaccines based on wild-type SARS-CoV-2 are being phased out of use, in line with WHO guidance [17].

**Figure 2:**
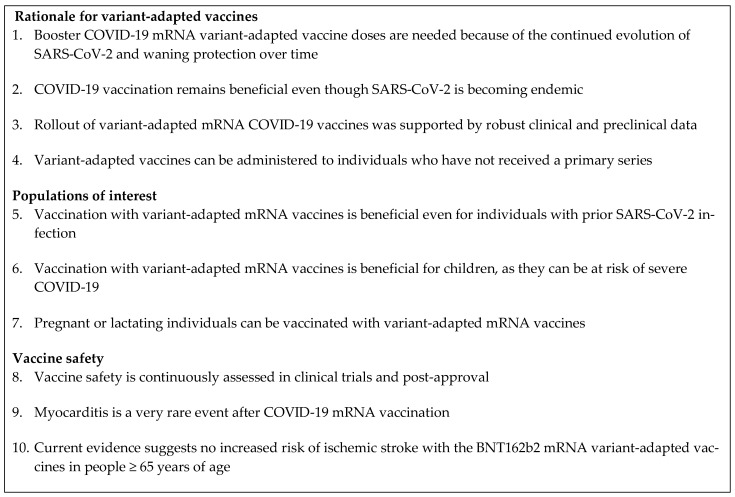
Responses to common misconceptions around COVID-19 mRNA variant-adapted vaccines covered in this review. COVID-19, coronavirus disease 2019; mRNA, messenger RNA; SARS-CoV-2, severe acute respiratory syndrome coronavirus 2.

This expert review focuses on responses to 10 common misconceptions regarding variant-adapted vaccines, relating to the rationale for these vaccines, populations of interest (people with prior SARS-CoV-2 infection, children, and pregnant or lactating individuals), and vaccine safety (Figure 2). This was not intended to be a systematic review of all misconceptions regarding variant-adapted vaccines. The review was informed by an initial comprehensive literature search, performed in PubMed and pre-print servers medRxiv and bioRxiv on 4 September 2023. Search terms included COVID-19, SARS-CoV-2, Omicron, vaccination, mRNA, bivalent, variant-adapted, efficacy, effectiveness, protection, seasonality, influenza, natural immunity, infection, children, pediatric, pregnancy, lactation, neonatal, myocarditis, cardiovascular, safety, adverse effect, risk, and pharmacovigilance. Additional publications, including those known to the authors, were included on a case-by-case basis following the initial literature search, owing to the rapidly evolving nature of this topic.

## 2. Rationale for COVID-19 mRNA Variant-Adapted Vaccines

**Figure 3:**
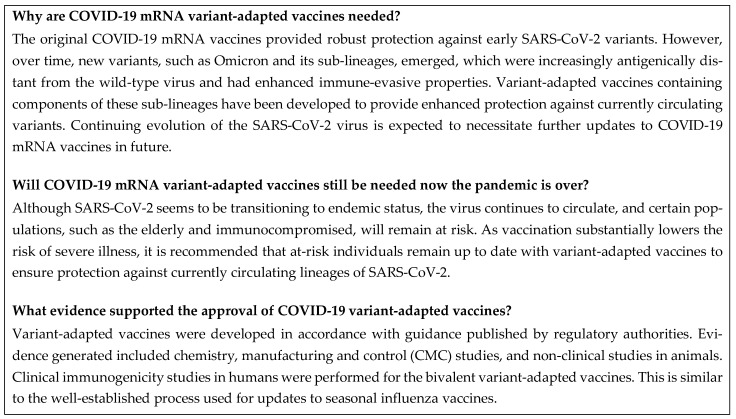
Frequently asked questions: rationale for variant-adapted vaccines. CMC, chemistry, manufacturing, and control; COVID-19, coronavirus disease 2019; mRNA, messenger RNA; SARS-CoV-2, severe acute respiratory syndrome coronavirus 2.

A summary of this section is shown in Figure 3.

### 2.1. Annual COVID-19 mRNA Variant-Adapted Vaccines Are Needed Because of Continued Evolution of SARS-CoV-2 and Waning Protection over Time

The original BNT162b2 and mRNA-1273 vaccines elicited robust protection against all circulating SARS-CoV-2 variants prior to the emergence of Omicron, with regard to symptomatic infection, severe disease, and death [18,19,20,21,22,23,24]. Individuals vaccinated with the original COVID-19 BNT162b2 or mRNA-1273 mRNA vaccines remained at lower risk of symptomatic disease and severe outcomes of COVID-19 infection vs. unvaccinated individuals in the periods during which Omicron variants circulated [25].

A third dose of original vaccine increased protection against symptomatic disease, hospitalization, and death caused by early Omicron sub-lineages, such as BA.1 and BA.2 [25,26]. Individuals with hybrid immunity derived from vaccination and prior infection were shown by several studies to have a high magnitude and durability of protection against re-infection and COVID-19 hospitalization [27,28]. However, as Omicron sub-lineages had partial immune escape mechanisms [29], antibodies elicited by original COVID-19 vaccines or by natural infection had reduced neutralizing activity against Omicron sub-lineages compared with the wild-type virus [8,29,30]. With 54 mutations, half of which affect the spike protein, some consider Omicron to be a distinct SARS-CoV-2 serotype [7]. Breakthrough infections subsequently became more common with the emergence of the Omicron variant and its sub-lineages [23,31,32,33]. In addition, both natural and vaccine-induced immunity wane over time [23,34].

By the time the Omicron variant emerged, immunity from the primary vaccination series had decreased for many individuals, leaving them susceptible to re-infection, albeit still with a reduced risk of severe disease, hospitalization, and death due to T-cell immunity being sustained to some degree [23,35]. Furthermore, the Omicron variant was highly transmissible and spread rapidly [6]. Consequently, infection rates during Omicron waves rose, increasing the chance of vaccinated individuals being exposed to a variant that was antigenically distinct from that included in the original vaccines. Over time, further Omicron sub-lineages emerged that were antigenically more distant from BA.1 and wild-type virus [8], and that had enhanced immune-evasive properties [29,36]. This necessitated the development of bivalent variant-adapted mRNA vaccines containing components of these sub-lineages in addition to the wild-type component.

**Figure 4:**
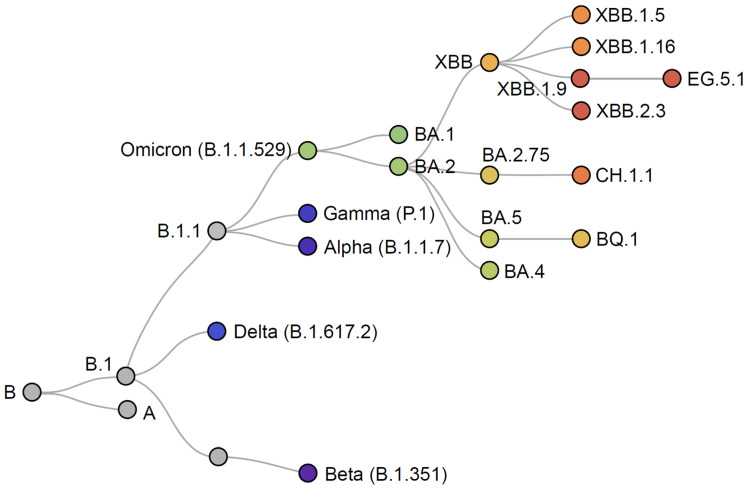
Phylogenetic relationships of SARS-CoV-2 variants. Adapted from Nextstrain [45].

Data from real-world studies supported the effectiveness of the BNT162b2 and mRNA-1273 bivalent vaccines against symptomatic infection and severe outcomes with BA.4 and BA.5, with similar levels of protection provided against the later sub-lineages XBB and XBB.1.5 [37,38,39,40,41,42,43]. Nevertheless, ongoing evolution and increasing immune evasiveness led to a requirement for further vaccine adaptation for autumn/winter 2023/2024. In addition, waning effectiveness of the bivalent BNT162b2 and mRNA-1273 Original/Omicron BA.4–5 vaccines has been observed from 2 to 4 months post-vaccination [44], likely due to the increasing antigenic difference between the vaccine and recent circulating sub-lineages, such as XBB.1.5 and XBB.1.16 (Figure 4) [45,46], and the increasing immune evasiveness of these sub-lineages.

Consequently, the WHO Technical Advisory Group on COVID-19 Vaccine Composition (TAG-CO-VAC) recommended that updated COVID-19 mRNA vaccines for the Northern Hemisphere autumn 2023/winter 2024 season should contain an XBB component [17]. Subsequently, the US Food and Drug Administration (FDA) Vaccines and Related Biological Products Advisory Committee and the European Medicines Agency (EMA) both recommended XBB-containing vaccines for autumn 2023 vaccination campaigns, with a preference for an XBB.1.5 component [13,14]. This decision was made to better match vaccines with current dominant variants and to provide improved protection.

The magnitude and durability of protection of a COVID-19 mRNA vaccine is dependent on the currently circulating variant, as increasing antigenic distance between the vaccine variant and circulating variants leads to reduced effectiveness. As the XBB.1.5 vaccines are antigenically better matched, they are expected to provide greater and more durable protection than the original vaccines against current variants [13,14,17]. Preclinical data suggested that XBB.1.5-containing vaccines elicit higher neutralizing antibody responses to currently circulating SARS-CoV-2 variants, compared with existing vaccines [47,48,49]. These vaccines have now been approved by the FDA and EMA [50,51,52]. Real-world and clinical studies have since shown that the monovalent XBB.1.5 vaccines result in potent neutralizing antibody responses against several variants, including XBB.1.5, XBB.1.16, BA.2.86, EG.5.1, FL.1.5.1, HV.1, HK.3, and JD.1.1 [53,54,55,56]. An early effectiveness study in Denmark has indicated a high level of protection from the XBB.1.5 vaccines [57].

A monovalent approach for the XBB.1.5 vaccines was recommended [13,14,58]. The original COVID-19 BNT162b2 and mRNA-1273 mRNA vaccines were monovalent, containing a component based on the spike protein of the wild-type SARS-CoV-2 virus. Initial studies of Omicron-adapted mRNA vaccines then revealed that, in vaccine-naïve individuals, a monovalent Omicron BA.1 vaccine had a lower neutralizing response to the ancestral strain compared with BA.1 [59]. As early VOCs, such as Delta, continued to circulate at this time, a bivalent approach was taken to maintain protection against these early VOCs while improving protection against emerging Omicron sub-lineages [60]. However, available sequencing data now indicate that the wild-type virus and antigenically related early VOCs, such as Alpha, Beta, Gamma, and Delta, are no longer circulating in humans.

In addition, the wild-type virus vaccine component elicits undetectable or very low levels of neutralizing antibodies against currently circulating variants, including XBB descendant lineages. Therefore, removal of the wild-type component is not expected to affect vaccine efficacy or effectiveness [17]. For this reason, the original COVID-19 monovalent vaccines based on the wild-type virus are no longer authorized for use in the USA [61].

The need to update COVID-19 vaccine composition is somewhat similar to observations for other viruses with high mutation rates, such as influenza, for which annual vaccine updates are typically required [62]. Influenza and other human coronaviruses typically follow a seasonal pattern, peaking in winter [62,63,64]. SARS-CoV-2 disease activity occurs year-round, but also follows a similar seasonal pattern, peaking between November and April [65,66,67]. Given the signs of waning of vaccine-induced immunity and evidence supporting improved protection with better-matched vaccines, periodic updates to the composition of COVID-19 mRNA vaccines are anticipated to be required to maintain vaccine effectiveness. The concept of better-matched vaccines has been well established with influenza, whereby vaccines are adapted annually in each hemisphere to antigenically match circulating strains to elicit higher effectiveness and protection. The WHO convenes technical consultations in February and September each year to recommend strains for inclusion in seasonal influenza vaccines in the Northern and Southern hemispheres, respectively [68].

### 2.2. COVID-19 Vaccination Remains Beneficial Even Though SARS-CoV-2 Is Becoming Endemic

Decreasing rates of COVID-19 mortality and severe outcomes in recent months suggest that SARS-CoV-2 is transitioning to endemic status, continuing to circulate but with more stable infection rates, in a similar way to seasonal influenza and other human coronaviruses [69]. In May 2023, the WHO declared that COVID-19 no longer constituted a public health emergency of international concern [70]. However, experience to date suggests that certain populations with susceptibility to severe outcomes, such as the elderly and immunocompromised, will remain at risk. SARS-CoV-2 disease activity occurs year-round, but similarly to other respiratory viruses [63,64,65,66,67], it peaks in autumn and winter. These peaks are likely to lead to a significant increase in infections, resulting in spikes in hospitalizations and deaths [65,66]. COVID-19 spans all age groups, including children in certain regions or countries [66]; thus, all populations can be affected. Those at high risk of severe outcomes include older adults and those with comorbidities [71,72,73], pregnant people [74], and the immunocompromised [75,76].

The WHO Strategic Advisory Group of Experts has advised prioritizing the future use of COVID-19 vaccines for populations at the greatest risk of death and severe disease, including older adults, people with underlying conditions, pregnant persons, children and adolescents, and frontline healthcare workers [66]. The EMA has issued similar advice [77]. Other populations, such as healthy adults, could also be at risk, depending on the future evolution of the virus. Post-acute sequelae of COVID-19 (PASC), or ‘long COVID’, can occur in healthy people, even those with relatively mild disease, and can have considerable long-term impact on everyday life [78,79]. Evidence from a systematic review and meta-analysis suggests that 45% of people with COVID-19 experience at least one unresolved symptom at 4 months post-infection, with fatigue, pain, impaired sleep, and breathlessness commonly reported [80]. Among people hospitalized for COVID-19, 45% had lasting changes in lung structure [80].

COVID-19 vaccination substantially lowers the risk of severe illness, hospitalization, and death from SARS-CoV-2 infection, and may reduce the severity and duration of PASC [37,38,39,40,41,42,81,82]. Variant-adapted mRNA vaccines that are better matched to current circulating variants than those based on previous variants are recommended for optimum protection [83]. Since the implementation of the bivalent variant-adapted vaccines, descendants of the recombinant variant XBB, including XBB.1.5 and XBB.1.16, have become dominant [84]. These later sub-lineages have growth advantages over earlier sub-lineages [12,85]. Owing to the increasing antigenic distance between the bivalent vaccine components and currently circulating lineages, as well as waning protection over time, vaccination with updated XBB-based variant-adapted vaccines is recommended in autumn 2023/winter 2024 to provide enhanced protection against XBB descendant lineages.

Although the impact on symptomatic disease and transmission was an additional benefit of COVID-19 vaccines during the early stages of the pandemic, owing to ongoing viral evolution, vaccination strategies with these updated vaccines are likely to focus on the prevention of severe disease [86]. As protection offered by natural infection or vaccination wanes over time, it is important to keep up to date with COVID-19 vaccinations.

### 2.3. Rollout of Variant-Adapted mRNA COVID-19 Vaccines Was Supported by Robust Clinical and Preclinical Data

The bivalent mRNA variant-adapted BNT162b2 and mRNA-1273 vaccines were developed in accordance with regulatory guidance that was published by the EMA and FDA in February and May 2021, respectively [87,88]. Evidence generated in line with this guidance supported regulatory approval and included data from chemistry, manufacturing, and control (CMC) studies, preclinical studies in animals, and clinical trials in humans, for both Original/Omicron BA.1 and Original/Omicron BA.4–5 vaccines.

Although the relationship between neutralizing antibodies and COVID-19 vaccine efficacy and effectiveness is not directly linear, as efficacy and effectiveness of the original COVID-19 mRNA vaccines had already been well established, immunogenicity data could be used as a surrogate endpoint for vaccine effectiveness [87,88]. This process of inferring vaccine efficacy through comparison of immune responses with those elicited by an existing authorized vaccine that has already been proven to be effective is known as immunobridging [89], and is similar to the process used for the regulatory approval of new seasonal influenza vaccines [90,91,92]. Because the vaccine platforms used for the bivalent vaccines were the same as those used for the original vaccines, the extensive safety profile of the original vaccines could be taken into account by regulatory authorities, along with safety assessments made during the immunobridging studies, mitigating the need for additional safety studies [87,88].

The clinical immunogenicity studies demonstrated that, compared with the original COVID-19 mRNA vaccines, the bivalent vaccines elicited improved neutralizing antibody titers against the Omicron variant contained in the vaccine, while maintaining neutralizing antibody activity against wild-type SARS-CoV-2 at a level similar to that of the original vaccines [93,94,95,96,97]. Based on these immunobridging studies and other preclinical, clinical, and CMC data generated, as well as the existing extensive safety database for the original monovalent COVID-19 vaccines, regulatory authorities determined that the bivalent vaccines met their stringent criteria for safety and effectiveness, and approved the vaccines for use in autumn 2022 [98,99].

The BA.4/BA.5 bivalent vaccines were initially authorized by the FDA for use as booster doses in August 2022 [100]; they were subsequently authorized for all doses administered, including primary series [61]. The EMA approved the bivalent BA.1 and BA.4/BA.5 vaccines for use as booster doses in September/October 2022 [101,102,103], and the UK Medicines and Healthcare products Regulatory Agency (MHRA) approved the bivalent BA.1 vaccines in August/September 2022, the BNT162b2 Original/Omicron BA.4–5 vaccine in November 2022, and the mRNA-1273 Original/Omicron BA.4–5 vaccine in February 2023 [104,105,106,107,108]. Post-approval, real-world data have confirmed the effectiveness of the mRNA bivalent variant-adapted vaccines against antigenically similar variants [37,38,39,40,41,42,109,110,111,112], demonstrating that the approval processes used were robust and appropriate.

For influenza, regular updates to seasonal vaccines and occasional development of pandemic vaccines are needed, owing to the high rate of antigenic drift of influenza viruses, which results in the continuing emergence of antigenically distinct strains [62]. The well-established process used for updates to seasonal influenza vaccines involves meetings of experts to determine which vaccine strains best match the viruses likely to be circulating in the upcoming season [113]. These meetings are convened by the WHO and occur annually for both Southern and Northern Hemisphere vaccine composition consideration. A similar antigen selection process is evolving for COVID-19 vaccines. The WHO TAG-CO-VAC convened in May 2023 to consider additional updates to COVID-19 vaccine composition, and will convene again approximately 6 months later [17]. The FDA met in June 2023 to discuss vaccine composition for autumn 2023 [13], and the EMA has indicated that recommendations on COVID-19 vaccine composition will be provided annually [14]. In future, it is likely that regulatory and public health authorities will advise vaccine manufacturers to revise the currently authorized vaccines annually, to ensure that they contain the most closely matched variant or variants to those circulating in the Northern Hemisphere autumn/winter season.

The EMA and FDA consider it acceptable to approve changes to the COVID-19 mRNA vaccine variant based on manufacturing/quality and non-clinical data primarily, provided that the vaccine platform has demonstrated predictability of clinical immunogenicity and reactogenicity [14,50]. As this has been clearly demonstrated for the original mRNA COVID-19 vaccines, it was possible for the monovalent XBB.1.5 vaccines to be approved based on CMC and non-clinical data [50]. CMC data include potency assays, stability/shelf-life data, and information on manufacturing consistency and process validation. CMC data can be used to support the approval of variant-adapted vaccines, as the manufacturing process, controls, and facilities are expected to be identical or very similar to that of the original vaccine [87,88]. Non-clinical data refer to immunogenicity data from relevant animal models demonstrating that the variant-adapted vaccine elicits a neutralizing antibody response to the vaccine variant and other relevant variants to a similar magnitude as the original vaccine [88]. Although clinical data in humans are being generated for the XBB.1.5 monovalent vaccines, this was not required for submission. This process is also similar to that used for updates to inactivated or recombinant protein seasonal influenza vaccines, for which additional clinical data specific to the new strain are not required [114].

As with influenza, basing approvals on CMC and preclinical data will allow for rapid evaluation of future variant-adapted COVID-19 vaccines, reducing the lag between variant emergence and vaccine composition, as well as ensuring that vaccines better match currently circulating dominant strains. Clinical immunogenicity studies for future variant-adapted COVID-19 vaccines are also planned, with data anticipated to be available by the time of vaccine implementation. Real-world data on vaccine effectiveness will be collected post-implementation.

### 2.4. Variant-Adapted Booster Vaccines Can Be Administered to Individuals Who Have Not Received a Primary Series

The bivalent variant-adapted BNT162b2 and mRNA-1273 vaccines were authorized for use as booster doses by major regulators, including the FDA and EMA [98,99,115], and were widely implemented around the world [116,117]. Booster doses of these vaccines were indicated regardless of the vaccine used for the primary vaccination course [83,98,115]. In the USA, the bivalent Original/Omicron BA.4–5 vaccines were also approved for use as a primary series [61]. The monovalent XBB.1.5 vaccines are approved as a single dose, irrespective of previous vaccination (with additional doses in children 6 months–4 years of age dependent on prior COVID-19 vaccination and infection history) [50,51,52].

EMA-recommended priority population groups for the monovalent XBB-based vaccines are older adults (>60 years of age), immunocompromised individuals, people with underlying medical conditions, pregnant individuals, and healthcare workers [14]. Populations eligible for vaccination are likely to differ by country; thus, healthcare providers should consult national guidance. Data will continue to be collected from clinical and real-world studies on administration of the XBB.1.5 monovalent vaccines to individuals with different COVID-19 vaccine histories.

## 3. Populations of Interest

**Figure 5:**
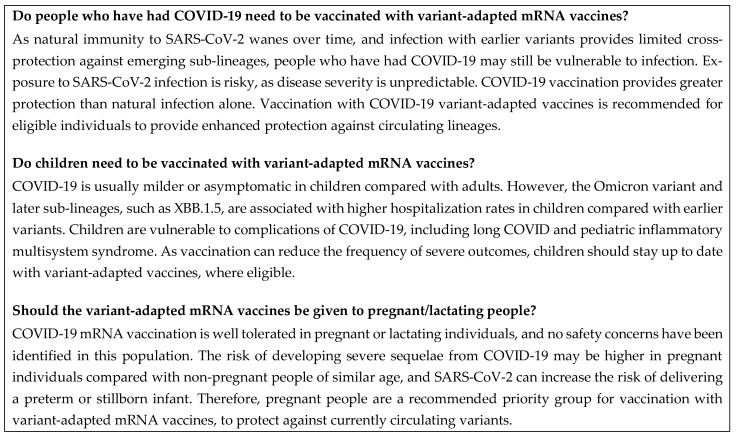
Frequently asked questions: populations of interest. COVID-19, coronavirus disease 2019; mRNA, messenger RNA; SARS-CoV-2, severe acute respiratory syndrome coronavirus 2.

A summary of this section is shown in Figure 5.

### 3.1. Vaccination with Variant-Adapted mRNA Vaccines Is Beneficial, Even for Individuals with Prior SARS-CoV-2 Infection

Natural immunity to SARS-CoV-2 wanes over time. Initial waning of antibodies to SARS-CoV-2 has been attributed to the loss of short-lived plasma cells, with a subsequent plateau in antibody levels due to the establishment of long-lived plasma cells [118]. Protection against re-infection may persist up to 40 weeks post-infection [119], but duration varies based on the circulating variant, the severity of the initial infection [120], age, and genetic factors [121,122,123]. In a meta-analysis of 91 studies, the overall risk of re-infection did not substantially differ by exposure risk or gender [124].

Natural immunity from infection with pre-Omicron variants provides limited cross-protection against the antigenically distant Omicron variants [8,30], particularly later sub-lineages [125,126]. Re-infection rates have risen in unvaccinated individuals since the emergence of Omicron [127]. Although protection against re-infection has been shown to remain high at 40 weeks post-infection with prior VOCs, protection against re-infection with BA.1 declines more rapidly (estimated at 36.1% after 40 weeks) [119]. Increased resistance to human interferons among emerging SARS-CoV-2 variants suggests a possible role for innate immunity, as well as resistance to neutralizing antibodies as immune evasiveness mechanisms [128].

Protection against re-infection provided by COVID-19 vaccination has been shown to be greater than that provided by natural infection alone. This is likely because the effect of COVID-19 vaccination on the production of SARS-CoV-2-specific immunoglobulin G antibodies is greater than that of natural infection [129,130]. Individuals with hybrid immunity, derived from vaccination and natural protection, have been shown to have a high magnitude and durability of protection against Omicron variants [27]. Therefore, vaccination is recommended even in individuals with prior infection. Variant-adapted mRNA vaccines containing Omicron components were recommended to provide protection against infection with Omicron variants and antigenically aligned sub-lineages [37,112,131], and variant-adapted vaccines containing XBB components are expected to provide enhanced protection against currently dominant lineages.

Exposure to SARS-CoV-2 infection is risky, as disease severity is unpredictable and long-term post-COVID-19 sequalae can occur even in healthy populations. Although protection provided by vaccination declines over time, protection against severe disease, hospitalization, and death from SARS-CoV-2 is maintained for longer than the protection against symptomatic infection [26,132,133,134]. Protection against severe outcomes provided by vaccination is greater than that provided by prior infection [122,135].

The effectiveness of the bivalent Omicron variant-adapted BNT162b2 and mRNA-1273 mRNA vaccines against severe disease has been demonstrated in several studies, including against antigenically aligned sub-lineages [37,39,40,41,42,109,110,112,136]. For example, the effectiveness of Omicron BA.4–5 vaccines against critical illness during the BA.4/BA.5 predominant period in adults in the USA was 61% at 7–89 days post-vaccination; corresponding effectiveness during XBB predominance was 58% [137]. However, vaccine-induced immunity against severe disease caused by currently circulating XBB descendent lineages begins to wane 2–4 months post-Original/Omicron BA.4–5 vaccination [44,137]. Factors influencing the decline of protection against severe outcomes with the bivalent vaccines include the immune evasion capabilities of emerging variants, such as BQ.1.1 and XBB.1.5, and the antigenic distance between these variants and Omicron BA.4/5 [46,109]. Therefore, vaccination with XBB-based variant-adapted vaccines is recommended for eligible at-risk individuals to maintain protection against severe outcomes of infection with currently circulating SARS-CoV-2 variants.

Although infections in vaccinated people can occur, in part due to the waning of antibody-mediated immunity over time and in part due to the emergence of newer and more immune-evasive variants, vaccinated individuals will have greater protection against severe disease than unvaccinated individuals if/when breakthrough infections occur. Symptomatic disease is likely to be milder in vaccinated individuals due to T-cell immunity. As of July 2023, almost 7 million deaths due to COVID-19 have been reported worldwide, including more than 2 million in Europe, 1 million in the USA, and 225,000 in the UK [11,138,139]. Vaccination remains the safest and most optimal form of protection against COVID-19-related symptomatic infection, hospitalization, and death [140].

### 3.2. Vaccination with Variant-Adapted mRNA Vaccines Is Beneficial for Children, as They Can Be at Risk of Severe COVID-19

Although the course of COVID-19 is usually milder or more often asymptomatic in children than in adults [141,142], data suggest there is variation in the rate of severe disease associated with Omicron sub-lineages, with infants and children <2 years of age bearing an increased proportion of severe illness [143]. Furthermore, Omicron sub-lineages have been associated with higher transmissibility than prior variants, resulting in high infection and hospitalization rates in pediatric populations [144], albeit still lower than those seen in adults [145].

In England, a three-fold increase in pediatric infections with Omicron sub-lineages vs. earlier variants was observed (from 40 to 120 admissions per day) [145], and, in the USA, a six-to-eight-fold increase in the incidence of infection in children <5 years of age was observed with Omicron vs. Delta (2.4–5.6 cases vs. 1.0–1.5 cases per 1000 persons per day) [144]. Pediatric admissions were higher during the BA.1 wave than with previous waves [146], and SARS-CoV-2 seroprevalence in pediatric populations substantially increased [147]. In addition, unlike previous variants, Omicron sub-lineages may manifest with febrile seizures in older children, although there is no evidence of any lasting clinical damage [148,149]. Children are also affected by newer emerging sub-lineages; the proportion of infections with the XBB.1.5 lineage was highest in children and adolescents 10–19 years of age compared with other age categories in the USA [150].

Approximately 25% of children and adolescents suffer from PASC, or ‘long COVID’ [151,152]. Furthermore, children infected with SARS-CoV-2 are vulnerable to developing a rare but severe hyperinflammatory complication, known as pediatric inflammatory multisystem syndrome temporally associated with SARS-CoV-2 (PIMS-TS) or multisystem inflammatory syndrome in children [153]. As many as 70% of patients with PIMS-TS require intensive care treatment [153]. More than 9000 cases of PIMS-TS and 70 deaths have been reported in the USA alone [154], although data from England suggest that the incidence of PIMS-TS is decreasing with successive Omicron sub-lineages [155].

The BNT162b2 and mRNA-1273 vaccines, including the bivalent variant-adapted vaccines, have been shown to reduce the number of infections and the frequency of severe outcomes in the pediatric population [156], with no safety signals other than rare events of myocarditis and pericarditis (see Section 4. Vaccine Safety) [157]. Vaccination may also strengthen community-level immunity, which is crucial for allowing schools to remain open, thus mitigating some of the indirect effects of COVID-19 on the well-being of children, including missed education and the resulting impact on academic achievement [158]. Therefore, children should stay up to date with variant-adapted vaccines, where eligible, to maintain protection as SARS-CoV-2 continues to evolve.

### 3.3. Pregnant or Lactating Individuals Can Be Vaccinated with Variant-Adapted mRNA Vaccines

COVID-19 vaccination of pregnant or lactating people is important to protect both parent and baby. Although the incidence rate of COVID-19 is similar for pregnant and non-pregnant people, pregnant individuals with symptomatic COVID-19 may be at higher risk of developing severe sequelae vs. non-pregnant, reproductive-aged individuals [74,159]. In addition, SARS-CoV-2 infection may increase the risk of delivering a preterm or stillborn infant, particularly in the third trimester [111,159,160,161]. Therefore, those who are pregnant are a recommended priority group for vaccination, along with elderly and immunocompromised individuals. Those who are pregnant, breastfeeding, trying to become pregnant, or who expect to become pregnant soon are encouraged to receive COVID-19 vaccines in line with recommendations [162]. Variant-adapted mRNA vaccines are recommended for protection against currently circulating variants [83].

Pregnant and lactating individuals generally have a comparable initial immunological response, including neutralizing antibody response and T-cell response, to COVID-19 vaccination to that observed in non-pregnant individuals [163,164,165]. Antibodies elicited by BNT162b2 and mRNA-1273 original vaccines are found in infant cord blood and the breast milk of vaccinated people, demonstrating that mRNA vaccines elicit a substantial antigen-specific immune response in the parent that is efficiently transferred to the fetus or newborn [166]. The bivalent variant-adapted vaccines have been shown to elicit robust neutralizing antibody responses to recent variants, including BA.5, BQ.1, and XBB.1.5, in maternal blood and umbilical cord blood [167]. The effectiveness of original COVID-19 mRNA vaccines in pregnant individuals and their infants is well established [168,169]. In line with this, the bivalent BNT162b2 and mRNA-1273 original/Omicron BA.4–5 vaccines have been shown to be effective against emergency department or urgent care encounters in immunocompetent pregnant persons. In a study of 191 pregnant people who received bivalent variant-adapted vaccine and 1701 unvaccinated pregnant people, the effectiveness of the vaccine during the period from September 2022 to May 2023 was 61% (95% CI: 22–81; median interval since vaccination 56 days) [137]. Maternal vaccination with COVID-19 mRNA vaccines has also been shown to provide protection against hospitalization of infants <6 months of age during a period when bivalent vaccines were being rolled out (although the number of pregnant people receiving the bivalent vaccine was small) [137,169]. Effectiveness of the monovalent XBB.1.5 vaccines in pregnant people will be monitored throughout rollout.

COVID-19 mRNA vaccination is well tolerated in pregnant or lactating individuals. Analyses from vaccine safety monitoring systems, including the v-safe After Vaccination Health Checker surveillance system, the v-safe COVID-19 Vaccine Pregnancy Registry, and the Vaccine Adverse Event Reporting System (VAERS), have not identified any safety concerns for pregnant people who received COVID-19 BNT162b2 or mRNA-1273 mRNA vaccines, or for their babies [170]. Vaccinated pregnant women did not report more frequent severe reactions than non-pregnant women, except for nausea and vomiting, which were slightly increased after the second dose [170]. In a prospective cohort study of breastfeeding women, no mother or infant experienced any serious AE post-BNT162b2 vaccination [171]. The safety of the monovalent XBB.1.5 vaccines in pregnant and lactating individuals and their babies will be monitored throughout rollout.

COVID-19 mRNA vaccination does not have an adverse effect on pregnancy outcomes. Data from a UK registry involving >24,000 individuals vaccinated prior to giving birth (90% with an mRNA vaccine) showed that the risk of stillbirth, low birth weight, and very or extremely premature birth was similar between vaccinated and unvaccinated individuals [172]. In a systematic review and meta-analysis of 23 studies including more than 117,000 vaccinated pregnant people, the majority of whom received mRNA vaccines, the risk of stillbirth was lower in the vaccinated cohort and there was no evidence of any increased risk of other adverse pregnancy outcomes with vaccination [173]. In another systematic review and meta-analysis of more than 80,000 people vaccinated against COVID-19 in pregnancy (98% with mRNA vaccines) and 250,000 unvaccinated pregnant people, vaccination was associated with a lower risk of neonatal intensive care unit admission and intrauterine fetal death [174]. A third systematic review found no evidence for an association between COVID-19 mRNA vaccines and fertility impairment [175]. A prospective cohort study in France and Switzerland detected no increased risk of congenital malformation in pregnant individuals exposed to COVID-19 mRNA vaccine in the first trimester vs. the second and third trimesters [176].

## 4. Vaccine Safety

**Figure 6:**
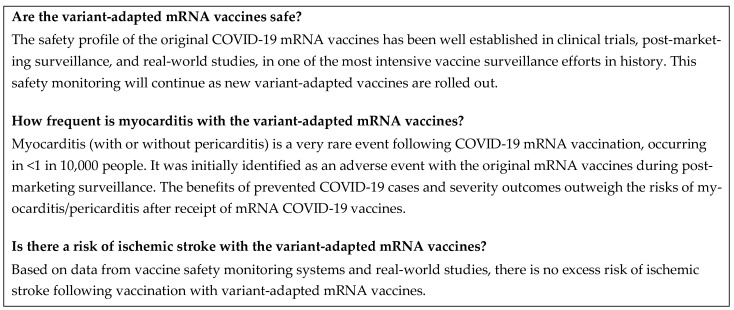
Frequently asked questions: vaccine safety. COVID-19, coronavirus disease 2019; mRNA, messenger RNA.

A summary of this section is shown in Figure 6.

### 4.1. Vaccine Safety Is Continuously Assessed in Clinical Trials and Post-Approval

The development of the original COVID-19 mRNA vaccines followed the same rigorous processes as for standard vaccine development, including non-clinical research, phase I to III clinical trials, and regulatory approval [3,177]. It was possible to expedite development via several methods, including the use of ongoing rolling regulatory submission processes, leveraging prior research and development from other infectious diseases, the utilization of novel vaccine technologies with shorter design-to-production times, rapid recruitment facilitated by participant willingness, high disease prevalence allowing a shorter time to demonstrate efficacy, and increased funding and collaboration. Every step of the regulatory pathway was fulfilled, including safety data requirements [3,177].

The COVID-19 mRNA vaccine trials enrolled very large numbers of participants; the pivotal phase II/III clinical trial of BNT162b2 included more than 43,500 individuals, and the mRNA-1273 trial included more than 30,000 individuals [178,179]. These trials demonstrated that COVID-19 mRNA vaccines are well tolerated, with mostly mild or moderate AEs [178,179]. However, they were not large enough to detect rare events, such as myocarditis, which occur in <1 in 10,000 vaccinated people [98,115]. Safety data collected within clinical trials are sufficient to characterize common AEs, such as the local and systemic reactions related to the immunogenicity of a vaccine, that occur within a short time after vaccination. Only after a vaccine is administered within large populations following licensure does it become possible to detect rare AEs that were not observed during clinical trials [180,181].

Post-authorization, the safety of COVID-19 vaccines has been monitored through multiple complementary passive and active surveillance systems [180,182], as well as an unprecedented number of real-world studies, including in children [157]. This enabled events such as myocarditis to be detected. Monitoring of COVID-19 vaccines has been described as one of the most intensive vaccine safety surveillance efforts in history [183], and has generated an unprecedented volume of data [184]. These surveillance databases cover extensive and heterogeneous populations, indicating that the data generated are robust. In addition, rapid cycle analysis has allowed tracking in near real-time, meaning that the risk of potential events can be detected and assessed in a timely manner [185]. As some systems and studies, such as the CDC’s V-safe, the EMA ACCESS (vACCine COVID-19 monitoring readinESS) project, apps for mobile reporting, and increased frequency of periodic safety update reports, were put in place during the COVID-19 pandemic [182,184,186], surveillance of COVID-19 vaccines is more extensive than that of other vaccines. This safety monitoring will continue as new variant-adapted vaccines are rolled out.

### 4.2. Myocarditis Is a Very Rare Event after COVID-19 mRNA Vaccination

Myocarditis (with or without pericarditis) is a very rare event following COVID-19 mRNA vaccination, occurring in <1 in 10,000 people [98,115]. Because of its rarity, it was only identified as an AE during the post-marketing surveillance period, after millions of vaccinations were administered globally [187,188]. The exact frequency varies depending on the mRNA vaccine used [189].

Myocarditis and pericarditis after COVID-19 BNT162b2 or mRNA-1273 vaccination can develop within just a few days, and have primarily occurred within 14 days [98,115]. Cases have been observed more frequently after the second vaccination [187]. Among patients in a large healthcare system in Israel, the incidence of myocarditis has been estimated at 2.13 per 100,000 people of all ages who received at least one mRNA vaccine dose [190]. Rates have consistently been reported to be higher in younger vs. older age groups (12–29 vs. ≥30 years of age), and in males vs. females [187,190,191,192]. Rates of myocarditis reported to VAERS as of June 2021 were 40.6 cases per million second doses in males 12–29 years of age and 2.4 cases per million second doses in males ≥30 years of age. Corresponding rates for females were 4.2 and 1.0 per million second doses, respectively [187]. The majority of reported cases in the literature have been mild to moderate in severity [187,190,191].

The clinical course and prognosis of post-vaccination myocarditis is comparable with myocarditis of other causes [193]. In fact, infection with SARS-CoV-2 is associated with myocarditis, and outcomes of infection-related myocarditis are generally more severe than myocarditis after vaccination. Data from the US CDC, VAERS, and a European observational study suggest an overall incidence of SARS-CoV-2 infection-related myocarditis of 15–400 per 10,000 people infected [194,195,196,197]. In a cohort study in four Nordic countries, myocarditis after BNT162b2 or mRNA-1273 vaccination was associated with better clinical outcomes within 90 days of hospital admission compared with myocarditis associated with COVID-19 infection [198]. A case series in England comprising more than 42 million individuals who received at least one BNT162b2 dose detected a higher risk of hospital admission or death from myocarditis after SARS-CoV-2 infection than after COVID-19 vaccination [199]. A retrospective cohort study in Hong Kong found a 92% lower mortality risk among individuals with myocarditis after BNT162b2 vaccination compared with those with viral infection-related myocarditis [200]. In a French nationwide study, the frequency of intensive care unit admission, mechanical ventilation, and death was lower in patients with post-BNT162b2 or mRNA-1273 vaccination myocarditis than in unvaccinated patients with myocarditis from any cause [201].

Magnetic resonance imaging patterns of myocardial injury with vaccine-associated myocarditis are similar to those seen with idiopathic or viral myocarditis, such as that induced by SARS-CoV-2, but with less severe abnormalities and less frequent septal involvement [202,203]. The clinical course of myocarditis and pericarditis post-vaccination is mild and similar to myocarditis or pericarditis in general [204]. For most cases of myocarditis and pericarditis post-mRNA vaccination, patients responded well to standard-of-care treatment and rest, and their symptoms improved [204], with a relatively short duration of hospitalization [189]. Some studies have suggested that post-vaccination myocarditis may be inflammatory in nature [205], leading to some patients being treated with non-steroidal inflammatory drugs [187]. Follow-up assessments of individuals with post-COVID-19 vaccination myocarditis after an average of 5.8 months demonstrated complete resolution, with a reduction in overall late gadolinium enhancement [206].

After reviewing available data on the risks and benefits of COVID-19 vaccination, the EMA and FDA have determined that the benefits of prevented COVID-19 cases and severity outcomes outweigh the risks of myocarditis/pericarditis after the receipt of mRNA COVID-19 vaccines. Regulatory bodies continue to recommend COVID-19 mRNA vaccines for the prevention of serious illness or death from COVID-19 [207,208].

### 4.3. Current Evidence Suggests No Increased Risk of Ischemic Stroke with the BNT162b2 mRNA Variant-Adapted Vaccines in People ≥ 65 Years of Age

The CDC Vaccine Safety Datalink (VSD) is one of several systems that monitor the safety of vaccines in the USA [209]. The Rapid Cycle Analysis (RCA) method in VSD allows population-based monitoring of potential outcomes associated with a vaccine in near real-time by examining outcome rates in recently vaccinated individuals during risk intervals in relation to rates during comparison intervals. Any association identified by RCA is considered to be a statistical signal that requires additional analytical investigation [210]. In January 2023, the CDC announced investigation of a preliminary signal for ischemic stroke, detected by the VSD RCA in a small number of individuals ≥65 years of age during the first 21 days after vaccination with BNT162b2 bivalent Original/Omicron BA.4–5. No other safety monitoring system has detected a signal, and it was thought unlikely that the VSD signal presented a true clinical risk [211].

The statistical signal slowly decreased over 8 weeks, only intermittently meeting signaling criteria. Findings also suggested a reduced rate of stroke in recipients of the bivalent vaccine vs. non-recipients during the comparison interval of 22–42 days post-vaccination. This temporal clustering during the risk and comparison intervals is difficult to interpret [212]. Confounding factors to consider are the small number of cases, as well as the concurrent administration of a high-dose or adjuvanted influenza vaccine on the same day as the bivalent vaccine in most cases. Additional considerations include the possibility of SARS-CoV-2 infection prior to vaccination, and the lack of follow-up time for the mRNA-1273 vaccine caused by distribution delays [212].

No evidence of excess risk of stroke has been detected in real-world studies. A nationwide analysis of patient electronic health records in the USA showed no significant difference in the risk of ischemic stroke between the BNT162b2 and mRNA-1273 bivalent vaccines. The risk was lower for the BNT162b2 bivalent vaccine compared with the original BNT162b2 vaccine booster at 1–21 and 22–42 days post-administration [213]. A large-scale retrospective safety analysis in Israel did not detect any indication of elevated risk of ischemic stroke following the administration of an original or bivalent BNT162b2 vaccine in individuals ≥60 years of age [214]. A study in France found no increased risk of cardiovascular events, including ischemic stroke, among recipients of a bivalent BNT162b2 Original/Omicron BA.4–5 vaccine compared with those who received the original BNT162b2 vaccine [215]. A study in England showed no evidence of an increased risk of stroke in the 21 days after vaccination with the BNT162b2 or mRNA-1273 bivalent Original/Omicron BA.1 vaccines in adults ≥50 years of age [216].

No excess risk of ischemic stroke has been reported by other vaccine safety monitoring systems, including the VAERS, the Centers for Medicare and Medicaid Services database, the Veterans Administration database, and databases in European countries. BioNTech/Pfizer have noted no increase in signal in their global safety database when comparing the original vaccine to the bivalent vaccines [217]. A formal epidemiological study is being initiated by the FDA to prepare for potential co-administration of COVID-19 and influenza vaccines in 2023–2024 [217].

## 5. Conclusions

Variant-adapted mRNA vaccines are key to protecting against emerging new variants of SARS-CoV-2. Addressing misconceptions and disinformation around these vaccines through the provision of scientific evidence can reassure potential vaccine recipients, potentially improving vaccination uptake and contributing to the ongoing management of COVID-19.

In the endemic phase of SARS-CoV-2, which is marked by a year-round incidence with seasonal peaks of disease activity, continuous monitoring of the evolution of the virus is needed in order to predict the dominant circulating variant for the next Northern Hemisphere autumn and winter season vaccination campaign. Vaccine variant selection based on this surveillance will be established in subsequent years to ensure that future variant-adapted vaccines are antigenically better matched to circulating variants and potential phylogenetically related future variants that may arise. The vaccination of at-risk populations during the peak activity season will be essential to ensure protection against hospitalization and other severe outcomes of COVID-19. Continued monitoring of vaccine safety through national and global surveillance databases is pivotal and will continue to be performed. Ongoing research on vaccination in special populations, such as older adults, people with comorbidities, immunocompromised people, and pregnant and lactating individuals, will also be of value. As the evolution of SARS-CoV-2 is dynamic, the continual assessment of viral fitness will be important to ascertain the impact of emerging variants on disease severity and transmission, especially in at-risk groups.
